# Favorable Short-Term Outcomes of a Patient With Symptomatic Bipartite Navicular Bone Treated by Screw Fixation

**DOI:** 10.7759/cureus.67959

**Published:** 2024-08-27

**Authors:** Takuji Yokoe, Takuya Tajima, Etsuo Chosa

**Affiliations:** 1 Orthopedic Surgery, Miyazaki University Hospital, Miyazaki, JPN

**Keywords:** subjective outcomes, nonunion, screw fixation, surgery, bipartite navicular

## Abstract

Bipartite navicular bone is an uncommon foot pathology that generally occurs in adolescent patients. However, some patients may become symptomatic during adulthood. When conservative treatment fails, surgical treatment is performed. However, due to a lack of high-quality evidence, the optimal surgical strategy for bipartite navicular remains unclear. The talonavicular arthrodesis and internal fixation of the fragment have been reported as the surgical choice of the symptomatic bipartite navicular. The internal fixation of the fragment may have a high risk of postoperative nonunion. However, talonavicular arthrodesis results in the limitation of the motion and function of the midfoot, which may cause dysfunction or osteoarthritis of the adjacent joints. This paper aims to present an adult case of symptomatic bipartite navicular that was treated by the internal fixation of the fragment using screws, with a favorable short-term outcome.

## Introduction

Bipartite navicular is a rare foot pathology [1,2]. In the relevant literature, bipartite navicular has been discussed as a differential diagnosis of nonunion, osteochondritis, or Mueller-Weiss disease and is clinically detected in teenagers [3-5]. However, some patients with bipartite navicular may become symptomatic in adulthood. To the best of our knowledge, there is only one report of the surgical treatment of bipartite navicular in an adult patient [6].

The optimal management of patients with symptomatic bipartite navicular remains unclear owing to the lack of high-quality evidence. In particular, few studies have reported the surgical management of symptomatic bipartite navicular. According to the published case reports [4-6], the authors performed surgery when conservative treatment failed to resolve the patient’s symptoms. The talonavicular arthrodesis and internal fixation of the fragment have been reported as promising surgical procedures for the symptomatic bipartite navicular. Chujo et al. have recently reported that talonavicular arthrodesis using a cannulated cancellous screw and compression screw resulted in good short-term outcomes in a 13-year-old male with bipartite navicular [5]. These authors reported that the internal fixation of the fragment may not be recommended because of the possibility of a high rate of nonunion. However, talonavicular arthrodesis prevents the motion and function of the midfoot, which would cause secondary dysfunction of the adjacent joints. At present, due to the limited number of surgically treated cases, whether internal fixation is an effective surgical option for symptomatic bipartite navicular remains a matter of debate.

We herein report an adult case of symptomatic bipartite navicular that was surgically treated by screw fixation, with a favorable short-term clinical outcome. Informed consent was obtained from the patient for the publication of this report and any accompanying images.

## Case presentation

**Figure 1:**
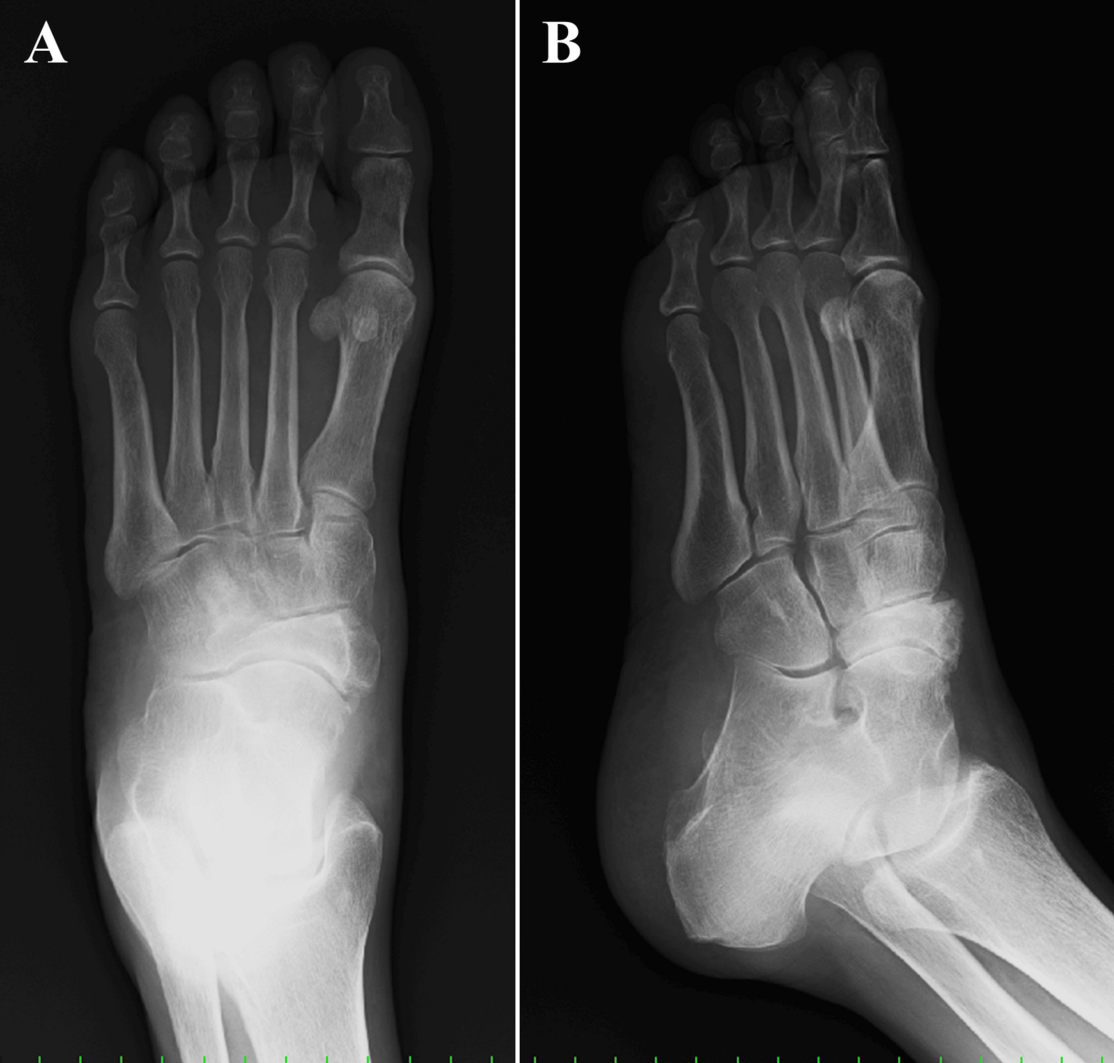
Preoperative plain radiographic findings. (A) Anteroposterior view and (B) internal oblique view.

**Figure 2:**
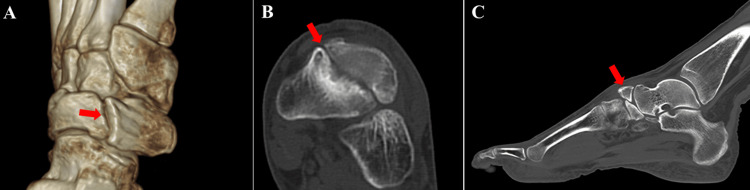
Preoperative computed tomography findings. The bipartite navicular was confirmed (red arrows). (A) 3D image, (B) axial view, and (C) sagittal view.

A 56-year-old female was referred to our department for the assessment and treatment of left medial midfoot pain. She was a nurse with a history of hypothyroidism and hypertension and had suffered from left foot pain and swelling, especially after working for several years. She visited a private orthopedic hospital and was diagnosed with a nonunion of the navicular bone. Three months of conservative treatment failed to resolve the patient’s symptoms. At the first presentation to our hospital, tenderness on palpation was detected in the navicular bone. Plain radiography and computed tomography (CT) revealed findings compatible with bipartite navicular (Figures 1, 2). The osteoarthritic change of the talonavicular joint was not detected on CT (Figure 2).

The preoperative patient-reported outcome measures (PROMs) including the foot and ankle outcome score (FAOS) [7], self-administered foot evaluation questionnaire (SAFE-Q) [8], and visual analog scale (VAS) scores are shown in Table 1.

**Table 1:** Pre- and postoperative patient-reported outcome measures. FAOS, foot and ankle outcome score; SAFE-Q, self-administered foot evaluation questionnaire; VAS, visual analog scale

	Preoperative	Postoperative
FAOS		
Symptoms	85.7	100
Pain	83.3	100
Function, daily living	89.7	98.5
Function, sports	75	90
Quality of life	43.8	100
SAFE-Q		
Pain and pain-related	72.2	94.4
Physical functioning	88.6	100
Social functioning	79.2	100
Shoe-related	83.3	100
General health	65	100
VAS score		
At rest	0/10	0/10
During walking	2/10	0/10

**Figure 3:**
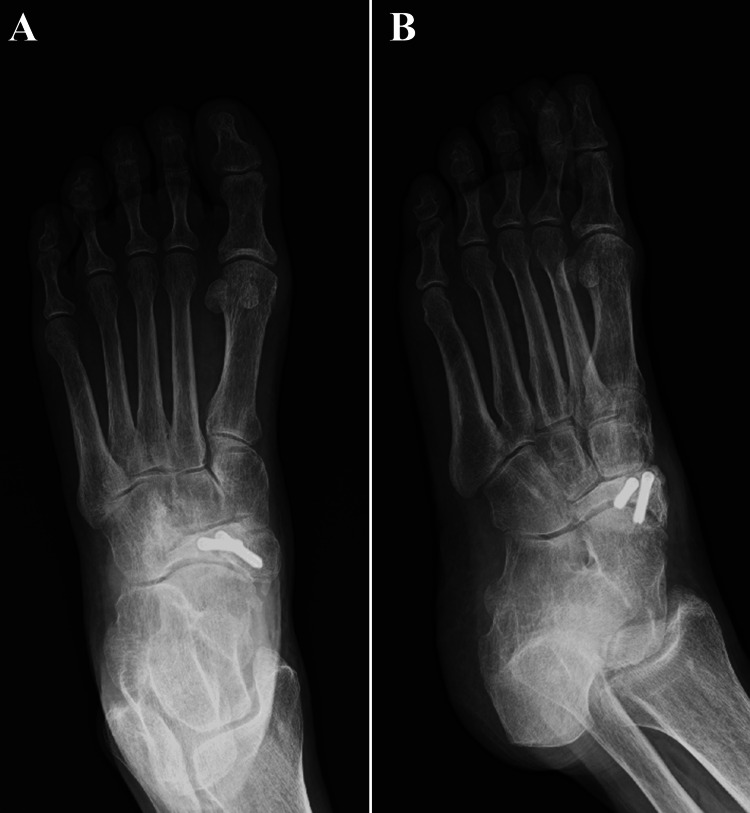
Postoperative plain radiographic findings. (A) Anteroposterior view and (B) internal oblique view.

Surgery was performed with the patient in the supine position on the operating table under spinal anesthesia. A thigh tourniquet was applied at 250 mmHg. Standard medial and dorsal incisions were used during surgery [9]. Via the medial incision, drilling was performed 10 times with a 1.5 mm Kirschner wire to accelerate the union of the bipartite navicular. No debridement or bone grafting was performed. Thereafter, through the dorsal incision, two fully threaded headless screws (Acutrak 2 Standard, Acumed, Hillsboro, OR) were applied under fluoroscopy. After lavage with saline, the surgical wounds were closed in a standard fashion. The postoperative radiography is shown in Figure 3.

**Figure 4:**
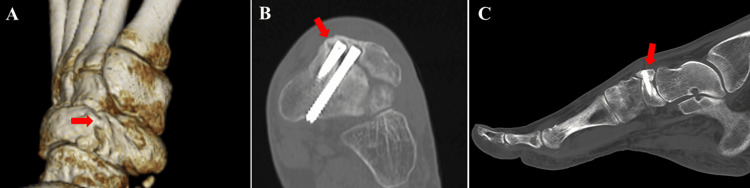
Postoperative computed tomography findings. The partial bone union of the medial side of the fragment was confirmed (red arrows). (A) 3D image, (B) axial view, and (C) sagittal view.

Postoperatively, immobilization was not performed on the operated foot. Ankle and finger range of motion (ROM) exercises were initiated from the first day after surgery, with no weight-bearing for four weeks. At four weeks after surgery, partial weight-bearing with a cane was allowed. Eight weeks after surgery, full weight-bearing was permitted. No wound complications nor infections were observed. At three months after surgery, return to work was permitted. The partial union of the bipartite navicular was detected on CT scans at 12 months after surgery (Figure 4).

At 18 months after surgery, there was no tenderness in the navicular bone. The postoperative FAOS, SAFE-Q, and VAS scores are shown in Table 1. She has worked as a nurse without any pain or swelling. The patient was very satisfied with the surgical outcome.

## Discussion

We herein presented an adult case of symptomatic bipartite navicular that was surgically treated by the internal fixation of the fragment, with a favorable short-term clinical outcome. There are few papers on bipartite navicular, and at present, the optimal management of patients with bipartite navicular has not been established [5,6]. As far as we know, only three papers have reported on the surgical management of patients with symptomatic bipartite navicular [4-6]. The majority of previous studies reported adolescent patients with symptomatic bipartite navicular [3,5]. However, some patients may become symptomatic, as shown in the present case and reported by Tanaka et al. [6]. Therefore, clinicians need to consider bipartite navicular as a differential diagnosis of medial midfoot pain in adult patients. Stress fracture, the nonunion of acute fracture, and Mueller-Weiss disease should be clinically suspected and discerned according to the involved location and imaging findings [3,10-14].

Regarding the management of symptomatic navicular, several authors performed surgical treatment after the failure of conservative treatment. The optimal surgical strategy remains unclear, as there are few studies reporting on the surgical management of bipartite navicular [4-6]. These authors recommended talonavicular arthrodesis as a surgical procedure for patients with bipartite navicular because of the high risk of postoperative nonunion. Yamaguchi et al. reported two cases of bipartite navicular that underwent internal fixation with screws: the first case by a threaded screw and the second case by two tapered threaded screws [4]. In the presented case, we used two tapered threaded screws that were greater in diameter and length than those used by Yamaguchi et al. [4]. We also inserted one screw as a bicortical screw. Partial bone union was confirmed on CT images at 12 months after surgery. Although the present case did not show complete union, partial union was observed on CT images at 12 months after surgery. However, the midfoot pain was completely resolved, and the postoperative subjective scores were favorable. This was similar to the cases of bipartite talus reported by Rose et al. [15]. The osteoarthritic change of the talonavicular or naviculo-cuneiform joint is an important factor when selecting the surgical procedure of the bipartite navicular. Tanaka et al. reported that an arthrodesis of the talar head and the medial fragment of the bipartite navicular and an arthrodesis of the lateral fragment of the bipartite navicular and the third cuneiform were performed using screws [6]. Given that talonavicular or naviculo-cuneiform osteoarthritic changes were not detected on preoperative CT images, we selected the internal fixation of the fragment because talonavicular arthrodesis would prevent the motion and function of the midfoot, which would result in the secondary dysfunction of the adjacent joints. However, the follow-up duration is relatively short, and careful observation is mandatory to clarify the efficacy of the internal fixation of the bipartite navicular.

## Conclusions

We presented an adult case of symptomatic navicular in which internal fixation was performed using two screws, with a favorable short-term outcome. In patients with a bipartite navicular, when the talonavicular joint does not show arthritic changes, internal fixation using large screws may be an effective surgical option to resolve the patient’s symptoms. However, careful long-term observation is mandatory to determine the long-term efficacy of this surgical procedure. In addition, further publications regarding the surgical management of the symptomatic bipartite navicular are needed because, to date, the optimal surgical strategy has not been established.

## References

[1] Wiley JJ, Brown DE (1981). The bipartite tarsal scaphoid. J Bone Joint Surg Br.

[2] Shawdon A, Kiss ZS, Fuller P (1995). The bipartite tarsal navicular bone: radiographic and computed tomography findings. Australas Radiol.

[3] Reade B, Atlas G, Distazio J, Kruljac S (1998). Mueller-Weiss syndrome: an uncommon cause of midfoot pain. J Foot Ankle Surg.

[4] Yamaguchi S, Niki H, Akagi R, Yamamoto Y, Sasho T (2016). Failure of internal fixation for painful bipartite navicular in two adolescent soccer players: a report of two cases. J Foot Ankle Surg.

[5] Chujo T, Nakasa T, Ikuta Y, Kawabata S, Adachi N (2023). Talonavicular arthrodesis using a screw and compression staple in a patient with bipartite navicular bone: a case report. Cureus.

[6] Tanaka Y, Takakura Y, Omokawa S, Kumai T, Sugimoto K (2006). Crank-shaped arthrodesis for a flatfoot with a bipartite navicular: a case report. Foot Ankle Int.

[7] Sierevelt IN, Zwiers R, Schats W, Haverkamp D, Terwee CB, Nolte PA, Kerkhoffs GM (2018). Measurement properties of the most commonly used foot- and ankle-specific questionnaires: the FFI, FAOs and FAAM. A systematic review. Knee Surg Sports Traumatol Arthrosc.

[8] Niki H, Tatsunami S, Haraguchi N (2013). Validity and reliability of a self-administered foot evaluation questionnaire (SAFE-Q). J Orthop Sci.

[9] Andrews NA, Patch DA, Torrez TW (2022). Which surgical approach is optimal for joint preparation in talonavicular fusion - a cadaver study. Foot Ankle Surg.

[10] Tosun B, Al F, Tosun A (2011). Spontaneous osteonecrosis of the tarsal navicular in an adult: Mueller-Weiss syndrome. J Foot Ankle Surg.

[11] Angthong C, Younger AS, Chuckpaiwong B, Harnroongroj T, Veljkovic A (2024). A novel update on the management of Müller-Weiss disease: presentation of a treatment algorithm. Cartilage.

[12] Shakked RJ, Walters EE, O'Malley MJ (2017). Tarsal navicular stress fractures. Curr Rev Musculoskelet Med.

[13] Mandell JC, Khurana B, Smith SE (2017). Stress fractures of the foot and ankle, part 2: site-specific etiology, imaging, and treatment, and differential diagnosis. Skeletal Radiol.

[14] Toren AJ, Hahn DB, Brown WC, Stone PA, Ng A (2013). Vascularized scapular free bone graft after nonunion of a tarsal navicular stress fracture: a case report. J Foot Ankle Surg.

[15] Rose B, Southgate C, Louette L (2013). Bipartite talus: a case series and algorithm for treatment. Foot Ankle Surg.

